# Prevalence, diagnostic methods, and clinical outcomes of wasting among paediatric cancer patients in Africa: A systematic review and meta-analysis

**DOI:** 10.1371/journal.pone.0353569

**Published:** 2026-07-09

**Authors:** Ivaan Pitua, Daisy Wannyana, Derrick Bary Abila, Felix Bongomin

**Affiliations:** 1 School of Medicine, College of Health Sciences, Makerere University, Kampala, Uganda; 2 Faculty of Health Sciences, Victoria University, Kampala, Uganda; 3 Uganda Child Cancer Foundation, Kampala, Uganda; 4 Uganda Cancer Institute, Kampala, Uganda; 5 Department of Medical Microbiology, Faculty of Medicine, Gulu University, Gulu, Uganda; Institute of Public Health from Guanajuato State, MEXICO

## Abstract

**Background:**

Malnutrition is a prevalent and modifiable co-morbidity in paediatric oncology, yet no comprehensive pan-African synthesis of its burden has been conducted. This systematic review and meta-analysis estimated the pooled prevalence of wasting and acute malnutrition among paediatric cancer patients in Africa, evaluated the impact of diagnostic assessment methods on reported prevalence, and characterised associations with adverse clinical outcomes.

**Methods:**

We searched PubMed/MEDLINE, EMBASE and Web of Science for observational studies published from January 2000 to December 2025 reporting wasting or acute malnutrition among children and adolescents (0–19 years) with confirmed malignancies in African healthcare settings. Two independent reviewers screened studies, extracted data, and appraised quality using the Joanna Briggs Institute Critical Appraisal Checklist. Pooled prevalence was calculated using a random-effects model with Freeman-Tukey double arcsine transformation. Heterogeneity was assessed using the *I*² statistic and Cochran’s Q test. Publication bias was evaluated with Egger’s regression test. Certainty of evidence was assessed using the GRADE framework.

**Results:**

Sixteen independent cohorts comprising 2,419 paediatric cancer patients across eight African countries were included. The pooled prevalence of wasting or acute malnutrition was 39.7% (95% CI: 30.7%–49.1%), with individual study estimates ranging from 11.7% to 67.6%. Between-study heterogeneity was substantial (*I*² = 95.3%; 95% CI: 92.7%–96.1%, p < 0.001). Studies using mid-upper arm circumference (MUAC) consistently detected substantially higher rates of malnutrition than those relying on weight-for-height or body mass index criteria from the same patient populations. No significant publication bias was detected (Egger’s test p = 0.503). GRADE certainty of evidence was very low, primarily due to heterogeneity. Wasting was independently associated with higher chemotherapy toxicity, treatment-related neutropenia, sepsis, and reduced overall survival across two of the included cohorts.

**Conclusions:**

Wasting and malnutrition affects about two in five children with cancer in the African countries studied. The diagnostic tool employed is the single most consequential determinant of detected prevalence, and weight-based metrics alone are inadequate in populations with large solid tumours. Universal adoption of MUAC-based screening, structured algorithm-guided nutritional intervention, and integration of socioeconomic vulnerability assessment into routine oncological care are evidence-based priorities for improving treatment tolerability and survival in this population.

## Introduction

Childhood cancer represents one of the most pressing yet under-recognized public health challenges in Africa, where survival rates remain markedly lower than those achieved in high-income countries [1,2]. While children diagnosed with cancer in North America and Western Europe now achieve five-year survival rates exceeding 80% [2], comparable figures in sub-Saharan Africa rarely surpass 30% [2–4]. This disparity reflects not only limited access to curative therapies but also a constellation of structural barriers that undermine treatment delivery, including inadequate supportive care infrastructure, delayed diagnosis, and the pervasive burden of malnutrition [5,6].

The World Health Organization Global Initiative for Childhood Cancer, launched in 2018, set an ambitious target to achieve at least 60% survival for children with cancer globally by 2030 [7]. Central to this goal is the recognition that survival cannot be improved through chemotherapy alone. The physiological capacity to tolerate intensive multimodal treatment is fundamentally compromised by malnutrition, yet this modifiable risk factor continues to receive insufficient attention in oncological care pathways across low- and middle-income countries [8,9].

Wasting, defined as low weight-for-height or low mid-upper arm circumference (MUAC), is an acute manifestation of malnutrition that signals immediate mortality risk [10]. In the context of pediatric oncology, wasting arises from a dual etiology. First, many African children present to oncology units already malnourished, reflecting widespread food insecurity and pre-existing growth faltering in resource-limited settings [11,12]. Second, the hypermetabolic and inflammatory state induced by cancer itself, compounded by the catabolic effects of chemotherapy, precipitates rapid somatic wasting even in children who were adequately nourished at diagnosis [13,14]. This phenomenon, known as cancer cachexia, is mediated by pro-inflammatory cytokines that drive skeletal muscle proteolysis and lipolysis, creating a syndrome distinct from simple starvation [15,16].

The biological consequences of wasting in pediatric cancer patients extend beyond energy depletion. Malnutrition impairs immune function, increasing susceptibility to severe infections, febrile neutropenia, and sepsis [17,18]. Furthermore, acute malnutrition alters drug pharmacokinetics, particularly for chemotherapeutic agents dosed by body weight or surface area, potentially leading to both under-dosing and heightened toxicity [8]. Malnutrition also disrupts gut mucosal integrity, exacerbating chemotherapy-induced mucositis and increasing translocation of enteric pathogens [19]. The cumulative effect is a cascade of treatment-related morbidity, prolonged hospitalization, treatment delays, and ultimately, reduced survival [20,21].

Despite these well-documented associations, the true magnitude of wasting among African children with cancer remains unclear. Existing prevalence estimates vary widely, ranging from below 10% to over 80% within different cohorts [22]. Many African oncology centers rely on weight-based metrics such as body mass index or weight-for-height Z-scores, which are unreliable in the presence of large solid tumors, organomegaly, or ascites [23,24]. Conversely, clinical assessment based on visible wasting alone has been shown to have a sensitivity of less than 45% compared to anthropometric measurement, meaning that more than half of wasted children are overlooked by visual inspection [23].

MUAC provides a direct measure of lean tissue mass in the upper arm and is less susceptible to confounding by tumor burden [25]. However, MUAC is not routinely incorporated into pediatric cancer assessment protocols across Africa, and national malnutrition screening programs often exclude children over five years of age, leaving adolescents with cancer particularly vulnerable to under-detection [21,26].

Beyond diagnostic inconsistency, a second major knowledge gap concerns the clinical outcomes associated with wasting in African pediatric cancer cohorts. While small single-center studies have reported associations between malnutrition and increased mortality [12,27], these findings have not been systematically synthesized.

By synthesizing data from multiple cohorts spanning diverse African settings, we aimed to provide the most comprehensive epidemiological picture to date of the malnutrition burden in pediatric oncology populations across the continent.

## Methods

### Protocol and registration

This systematic review and meta-analysis was conducted and reported in accordance with the Preferred Reporting Items for Systematic Reviews and Meta-Analyses (PRISMA) guidelines [28]. Full checklist can be accessed in additional files (S1 File). The protocol was prospectively registered in the PROSPERO International Prospective Register of Systematic Reviews (Registration Number: CRD420251237859) and published in PLOS ONE [29], with the review being conducted in accordance with the registered protocol.

### Information sources and search strategy

A comprehensive search strategy was developed using Medical Subject Headings (MeSH) and free-text keywords. The search was conducted in PubMed, EMBASE and Web of Science from January 1, 2000 to December 31, 2025. The core search combined terms for the population (paediatric, child, adolescent), condition (cancer, malignancy, neoplasm, leukaemia, lymphoma), exposure (wasting, cachexia, malnutrition, MUAC, BMI, weight-for-height), and setting (Africa, sub-Saharan Africa, and individual African country names). Reference lists of all included studies and relevant systematic reviews were manually screened to identify additional eligible studies. The full search string is provided in additional files (S2 File).

### Eligibility criteria

We included observational studies (cross-sectional, prospective cohort, retrospective cohort, and case-control designs) that reported the prevalence of wasting, cachexia, or acute malnutrition among children and adolescents aged 0–19 years with a confirmed diagnosis of any malignancy (haematological or solid tumour), receiving care at healthcare facilities located within Africa. Studies were eligible if they defined wasting or acute malnutrition using any recognised standard, including anthropometric Z-scores (weight-for-height/length, BMI-for-age, or MUAC-for-age below −2 SD), MUAC absolute thresholds, triceps skinfold thickness below the 5th percentile, clinician-assigned nutritional categories (poor/fair/good), or Subjective Global Assessment.

We excluded case reports and case series with fewer than ten patients, conference abstracts without sufficient quantitative data, qualitative studies, narrative reviews, editorials, and animal studies. Studies conducted outside Africa and those enrolling exclusively adult populations (aged ≥20 years) were also excluded.

### Study selection

**Fig 1:**
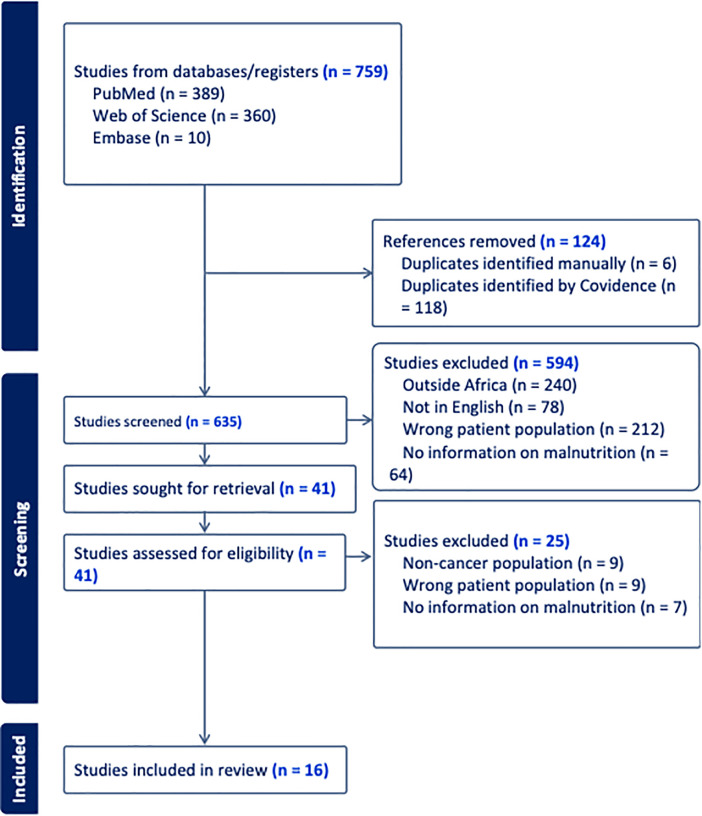
PRISMA flow diagram showing the screening process.

Search results were imported into Covidence systematic review software for deduplication and screening. Two independent reviewers (IP and DBA) screened all titles and abstracts for relevance. Full-text articles of potentially eligible studies were retrieved and assessed against the inclusion criteria. Disagreements at either stage were resolved by consensus. The study selection process is documented in a PRISMA flow diagram (Fig 1).

### Data extraction

Data were extracted independently by two reviewers using a standardised form piloted on five randomly selected studies. Extracted variables included: study citation and country; study design and data collection period; sample size; participant age (mean, median, or age strata); cancer types and proportions; method and tool of nutritional assessment; wasting definition and cut-off employed; number and proportion of patients classified as wasted or acutely malnourished; and, where reported, outcome data including mortality, neutropenia, sepsis, and overall survival. Where studies reported multiple nutritional metrics, all available wasting-specific data were extracted.

### Risk of bias assessment

The methodological quality of each included study was independently assessed by two reviewers using the Joanna Briggs Institute (JBI) Critical Appraisal Checklist for Prevalence Studies [30]. This nine-item instrument assesses the adequacy of the sampling frame, sample size justification, representativeness of subjects, use of standardised and validated measurement conditions, appropriateness of statistical methods, and management of missing data. Studies scoring 8–9 out of 9 were classified as low risk, 6–7 as moderate risk, and ≤5 as high risk of bias. Disagreements between the two reviewers (IP and DBA) were resolved by discussion; where consensus could not be reached, a third reviewer (FB) would adjudicate.

### Statistical analysis

A meta-analysis was performed using R Statistical Software (version 4.5.2) with the meta and metafor packages. The pooled prevalence of wasting was calculated using the Freeman-Tukey double arcsine transformation to stabilise variances, which is the recommended approach for pooling proportions that approach the boundaries of 0 or 1. A random-effects model was applied using the DerSimonian-Laird method, given the anticipated heterogeneity of healthcare settings, diagnostic methods, and patient populations across the African continent.

Between-study heterogeneity was quantified using the I² statistic, with values of 25%, 50%, and 75% representing low, moderate, and substantial heterogeneity respectively, and by Cochran’s Q test (with significance at p < 0.10). Publication bias was assessed by visual inspection of a contour-enhanced funnel plot and by Egger’s linear regression test (with significance at p < 0.05). Certainty of the body of evidence was evaluated using the Grading of Recommendations Assessment, Development and Evaluation (GRADE) [31] framework, assessing five domains: risk of bias, inconsistency, indirectness, imprecision, and publication bias. No formal subgroup analyses were pre-specified as primary analyses; exploratory interpretation of heterogeneity by diagnostic tool type and cancer category was conducted narratively based on the extracted study data.

## Results

### Study selection

The initial comprehensive database search across PubMed/MEDLINE, Web of Science and EMBASE yielded 759 records after deduplication. Following title and abstract screening, 41 studies were retrieved for full-text assessment. After full-text evaluation and the exclusion of overlapping clinical reports to prevent double-counting of patient populations, 16 independent studies (cohorts) were included in the final synthesis. The complete screening process is documented in the PRISMA flow diagram (Fig 1).

### Characteristics of included studies

The 16 included cohorts encompassed 2,419 paediatric cancer patients across eight African countries: South Africa (n = 5 cohorts), Malawi (n = 3), Uganda (n = 2), Ethiopia (n = 2), Kenya (n = 1), Ghana (n = 1), Tanzania (n = 1), and Morocco (n = 1). Sample sizes ranged from 40 to 463 patients per study. The majority of cohorts evaluated mixed paediatric oncology populations, while three focused exclusively on Wilms tumour and one on Hodgkin lymphoma. Study designs included prospective cohorts (n = 6), retrospective cohorts (n = 5), cross-sectional studies (n = 3), and observational studies (n = 2). Detailed characteristics of all included studies are presented in Table 1.

**Table 1:** Characteristics of the 16 studies included in the systematic review and meta-analysis.

Author (Year)	Country	Study Design	N	Age (Mean/Median, Range)	Cancer Types Studied (n, %)	Diagnostic Method & Wasting Definition	Wasted (n) & Prevalence (%)	Clinical Outcomes & Key Statistical Findings
**Schoeman et al. (2025) [32]**	South Africa	Prospective cohort	320	Strata: < 5 years and age ≥ 5 years	Mixed pediatric cancers (specific breakdown not specified in abstract)	Acute malnutrition via MUAC-for-age Z-scores < −2 SD and BMI-for-age Z-scores	78 (24.4%)	Stunting was significantly associated with poorer 3-year overall survival (HR 1.8). Wasting was significantly higher in older children age 5 years (18.7%) compared to < 5 years (3.9%).
**Israëls et al. (2008) [33]**	Malawi	Observational	128	Pediatric (specific mean not reported)	Mixed pediatric malignancies	MUAC or Triceps Skinfold Thickness (TSFT) < 5th percentile against 1978 NCHS curves	76 (59.4%)	Over half of patients were acutely malnourished by arm anthropometry (MUAC/TSF: 59.3%, AMA: 55.1%), confirming severe baseline deficits.
**Wilde et al. (2010) [34]**	Malawi	Retrospective cohort	40	Mean: 4.2 years	Nephroblastoma / Wilms tumour (n = 40, 100%)	“Poor” nutritional status by clinical judgement combined with BMI (mean BMI 15 kg/m²)	20 (50.0%)	Poor nutritional status contributed heavily to poor overall survival; the 1-year survival rate was exceptionally low, ranging between 25% and 53%.
**Shikuri et al. (2013) [35]**	Kenya	Cross-sectional	60	Range: 6 months – 14 years	Mixed pediatric solid tumors and hematological malignancies	Wasting defined by standard anthropometric parameters	7 (11.7%)	Nutrition impact symptoms (e.g., vomiting, decreased appetite) were significantly more pronounced in advanced stages II/III. Treatment type directly compromised food intake in 88.5% of the cohort.
**Salifu et al. (2024) [18]**	Ghana	Prospective cohort	133	Median: 4.5 years (Range: le 12 years)	Solid tumors (n = 81, 60.9%), Leukemias (n = 31, 23.3%), Lymphomas (n = 21, 15.8%)	Wasting defined as Upper Arm Muscle Area (UAMA) or MUAC le 5th percentile	32 (24.1%)	Wasting by MUAC/UAMA was significantly associated with a higher risk of anaemia and mucositis. 25% of all early deaths occurred exclusively in children wasted by MUAC.
**Nyeko et al. (2025) [23]**	Uganda	Prospective cohort	144	Range: 6 months – 17 years	Solid tumors (n = 89, 61.8%), Hemato-lymphoid (n = 55, 38.2%)	Anthropometric Z-scores compared directly against clinical “Visible Wasting”	57 (39.6%)	Wasted patients faced significantly higher risks for neutropenia (OR 3.63), sepsis (OR 4.50), and short-term mortality (OR 3.08). Clinical visible wasting missed 56.1% of anthropometrically verified wasted children.
**Geel et al. (2021) [36]**	South Africa	Retrospective cohort	271	Median: ~ 8.5 years	Hodgkin lymphoma (n = 271, 100%)	Defined as underweight/wasting via Weight-for-Age (WFA) <−2 SD	89 (32.8%)	HIV-infected patients were significantly more likely to present with wasting. 5-year overall survival (OS) of HIV+ patients was severely reduced at 49% vs 84% for HIV-uninfected.
**Draper et al. (2018) [37]**	South Africa	Prospective cohort	66	Median: 3.8 years	Nephroblastoma / Wilms tumour (n = 66, 100%)	Malnutrition defined rigorously by MUAC and TSFT criteria	38 (57.6%)	Significant positive relationship established between MUAC-defined malnutrition and treatment-induced neutropenia; malnourished patients experienced prolonged neutropenic episodes.
**Tola et al. (2023) [38]**	Ethiopia	Prospective obs.	73	Mean: 7.82 years (Range: 6 months – 16 years)	Mixed pediatric (ALL, AML, Wilms tumour, Hodgkin lymphoma)	Severe acute malnutrition (SAM) based on WHO clinical/anthropometric records	20 (27.4%)	SAM was the most prevalent comorbidity. The presence of any comorbidity (including SAM) drastically increased the risk of severe Grade 3/4 Adverse Drug Events (AOR 12.7).
**Yifru & Muluye (2015) [39]**	Ethiopia	Retrospective	71	Mean: 7 pm 4 years	Hematological (n = 43, 60.6%), Solid tumors (n = 28, 39.4%)	Concomitant presentation of stunting, wasting, and underweight	48 (67.6%)	High rates of concomitant malnutrition (>67%). Severe therapy interruptions and hospital delays were predominantly noted due to unaffordable/unavailable chemotherapy.
**Katabalo et al. (2025) [26]**	Tanzania	Cross-sectional	65	Strata: < 5 years, 5–10 years, > 10 years	Mixed pediatric cancers	Any objective anthropometric indicator (MUAC, WFA, HFA, BMI-Z, TSFT) below age-specific threshold	27 (41.5%)	78.4% of households were at risk of severe food insecurity. Undernutrition was statistically significantly more likely in children older than 5 years.
**Lifson et al. (2016) [40]**	South Africa	Prospective obs.	76	Pediatric (specific mean not reported)	Nephroblastoma / Wilms tumour (n = 76, 100%)	Any deficit identified via MUAC, TSFT, or weight/height	50 (65.8%)	Demonstrated that using weight in isolation significantly underestimated malnutrition due to tumor mass. Malnutrition on admission did not significantly predict early mortality in this cohort.
**Geddara et al. (2023) [27]**	South Africa	Retrospective cohort	139	Median: 4.3 years (Range: 0.5–11.3 years)	Solid tumors (n = 100, 71.9%), Hematological (n = 39, 28.1%)	WHO criteria utilizing Weight-for-height Z-scores (<5y) and BMI Z-scores (>5y)	24 (17.3%)	Wasting was higher in solid tumors (21.2%) vs hematological (7.7%), but not statistically significant (P = 0.24). Admission malnutrition did not significantly increase early mortality.
**Wannyana et al. (2025) [24]**	Uganda	Cross-sectional	270	Range: 2–17 years	Mixed pediatric cancers (with GI tract cancers specifically evaluated)	Wasting strictly defined by MUAC-for-age Z-scores < −2 SD	74 (27.4%)	Wasting prevalence was 20% higher in children ≥ 5 years (aPR 1.2; p = 0.002). Cancers near the GI tract (aPR 1.1) and treatment side effects (aPR 1.1) both significantly increased wasting risk by 10%.
**Tazi et al. (2008) [41]**	Morocco	Observational	100	Mean: 7 years (Range: 1–18 years)	Burkitt lymphoma (19%), AML (18%), ALL (14%), solid tumors (49%)	Z-scores calculated for MUAC, TSFT, Weight-for-Age (WFA), and BMI	39 (39.0%)	Nutritional deficits were identified significantly more frequently when utilizing arm anthropometry (MUAC/TSF) compared to weight-based metrics. Solid and CNS tumors exhibited greater deficits.
**Huibers et al. (2022) [21]**	Malawi	Retrospective cohort	463	63.5% of the cohort was age ≥ 5 years old	Mixed pediatric hematological malignancies and solid tumors	Severe Acute Malnutrition (SAM) and Moderate Acute Malnutrition (MAM) via anthropometry	293 (63.3%)	Malnutrition was significantly more common in children ≥ 5 years (70.0%) compared to < 5 years (51.8%; p < 0.0001). Presence of SAM significantly increased overall mortality risk (HR 1.6; 95% CI 1.1–2.3, p = 0.012).

MUAC = mid-upper arm circumference; TSFT = triceps skinfold thickness; WFA = weight-for-age; WFH = weight-for-height; BMI = body mass index; SAM = severe acute malnutrition; MAM = moderate acute malnutrition; UAMA = upper arm muscle area; NR = not reported.

### Risk of bias

The overall methodological quality of included studies was moderate to high. JBI scores ranged from 6/9–9/9. Six studies were rated low risk of bias (score 8–9/9. The remaining ten were rated moderate risk of bias (score 6–7/9). Common methodological limitations in moderate-risk studies included the absence of sample size justification, reliance on retrospective data with incomplete records, and variability in the documentation of nutritional assessment procedures. No study was rated high risk of bias. Full risk of bias scores are presented in Table 2.

**Table 2:** Risk of bias assessment using the JBI critical appraisal checklist for prevalence studies.

Study (Author, Year)	Study design	JBI score (out of 9)	Overall risk of bias
Schoeman et al. (2025) [32]	Prospective cohort	8/9	Low
Israëls et al. (2008) [33]	Observational	7/9	Moderate
Wilde et al. (2010) [34]	Retrospective cohort	6/9	Moderate
Shikuri et al. (2013) [35]	Cross-sectional	7/9	Moderate
Salifu et al. (2024) [18]	Prospective cohort	8/9	Low
Nyeko et al. (2025) [23]	Prospective cohort	9/9	Low
Geel et al. (2021) [36]	Retrospective cohort	6/9	Moderate
Draper et al. (2018) [37]	Prospective cohort	8/9	Low
Tola et al. (2023) [38]	Prospective observational	7/9	Moderate
Yifru & Muluye (2015) [39]	Retrospective	6/9	Moderate
Katabalo et al. (2025) [26]	Cross-sectional	7/9	Moderate
Lifson et al. (2016) [40]	Prospective observational	8/9	Low
Geddara et al. (2023) [27]	Retrospective cohort	6/9	Moderate
Wannyana et al. (2025) [24]	Cross-sectional	8/9	Low
Tazi et al. (2008) [41]	Observational	7/9	Moderate
Huibers et al. (2022) [21]	Retrospective cohort	7/9	Moderate

### Certainty of evidence

The certainty of the body of evidence was evaluated using the GRADE framework. Because all included studies were observational, the baseline certainty began at “Low.” The evidence was downgraded by one additional level due to serious inconsistency, driven by the high and unexplained statistical heterogeneity (I² = 95.3%). No downgrades were applied for risk of bias, indirectness, imprecision (the total sample of 2,419 provided narrow confidence intervals), or publication bias. The final GRADE certainty rating was Very Low (Table 3).

**Table 3:** GRADE evidence profile for the pooled prevalence of wasting and acute malnutrition.

Outcome	No. studies (N)	Risk of bias	Inconsistency	Indirectness	Imprecision	Publication bias	Certainty
Prevalence of Wasting / Acute Malnutrition	16 studies (N = 2,419)	Not serious	Serious ↓	Not serious	Not serious	Not serious	VERY LOW ⊕◯◯◯

Downgraded one level (↓) due to high statistical heterogeneity (I² = 95.3%, p < 0.001).

### Pooled prevalence and publication bias

**Fig 2:**
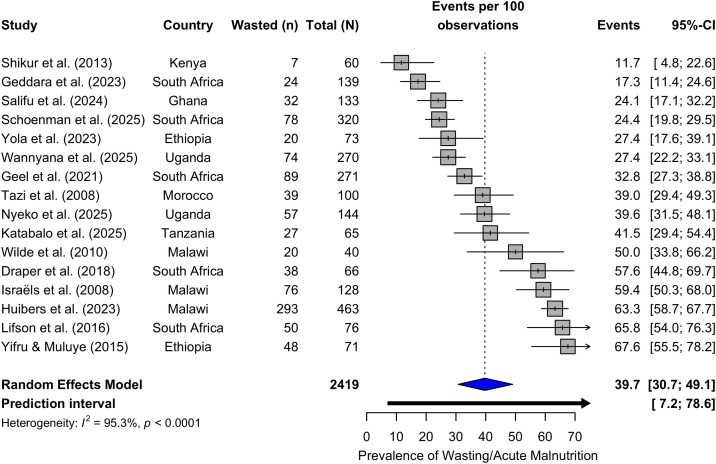
Forest plot showing the pooled prevalence of wasting and acute malnutrition across 16 African paediatric cancer cohorts.

The prevalence of wasting and acute malnutrition at diagnosis or hospital admission varied substantially across included studies, ranging from a low of 11.7% to a high of 67.6%. Data from the 16 independent cohorts (N = 2,419) were pooled using a random-effects model with logit transformation. The overall pooled prevalence of wasting among paediatric cancer patients in Africa was 39.7% (95% CI: 30.7%–49.1%; Fig 2).

**Fig 3:**
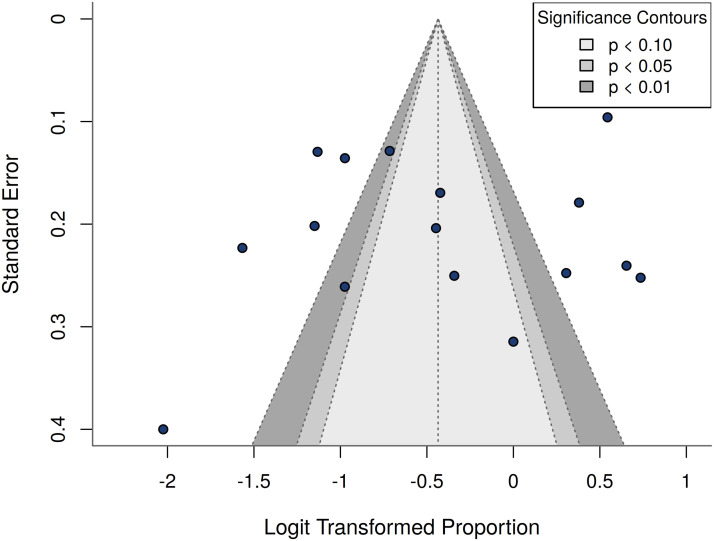
Contour-enhanced funnel plot assessing publication bias.

A high degree of between-study heterogeneity was observed (I² = 95.3% [95% CI: 92.7%–96.1%]; Cochran’s Q p < 0.0001), consistent with the diverse geographic regions, differential healthcare infrastructure, and varying diagnostic criteria employed across the included studies. Visual inspection of the contour-enhanced funnel plot revealed a symmetrical distribution of included studies, suggesting the absence of meaningful small-study effects (Fig 3). This was statistically confirmed by Egger’s linear regression test (bias coefficient = −2.13; SE = 3.09; t = −0.69; df = 14; p = 0.503), indicating no significant publication bias.

## Discussion

This study provides the first comprehensive pan-African synthesis of the burden of wasting and acute malnutrition among children with cancer, revealing that approximately two in five children diagnosed with cancer in Africa are acutely malnourished at presentation or during treatment. The pooled prevalence of 39.7% substantially exceeds estimates from high-income settings, where wasting prevalence in pediatric oncology populations typically ranges between 6% and 15% [21,32,42–44]. This disparity shows the unique convergence of pre-existing food insecurity, delayed cancer diagnosis, and limited supportive care resources that characterizes pediatric oncology care across much of sub-Saharan Africa [5,22].

A key finding of this review is that the diagnostic tool employed is the single most consequential determinant of detected wasting prevalence. Studies using mid-upper arm circumference systematically identified substantially higher rates of malnutrition compared to those relying on weight-for-height Z-scores, visual or body mass index criteria within the same patient populations. For example, in a Ugandan cohort, visible wasting had low sensitivities in detecting wasting generally (43.9%) and moderate wasting (19.4%) compared to the anthropometric measures, specifically, with false negative rates of 56.1% and 80.6%, respectively [23]. These findings are consistent with mechanistic understanding of how tumor mass can artificially inflate body weight. It is important to note, however, that these assessment methods are not interchangeable: MUAC, BMI-based indices, weight-for-height, triceps skinfold thickness (TSFT), and clinician-assigned categories each capture a distinct dimension of nutritional status, ranging from lean tissue mass to overall body proportionality, and this methodological heterogeneity likely contributes substantially to the variability in pooled prevalence estimates observed in this review. TSFT, used in two included cohorts [41,45] as a component of the wasting/malnutrition definition, was not evaluated as an independent predictor of clinical outcomes in any included study; given its potential to detect fat mass depletion distinct from the lean mass captured by MUAC, this represents a specific gap for future research.

Weight-based metrics alone are inadequate for nutritional screening in populations with high prevalence of solid tumors and thus the integration of MUAC into routine oncological triage protocols is a fundamental requirement for case detection. MUAC measurement is rapid, requires minimal training, and can be performed reliably by nurses or community health workers using inexpensive, reusable tapes [25]. However, MUAC-based screening also carries an important limitation: WHO reference cut-offs for MUAC are validated only for children aged 6–59 months, and although several MUAC-for-age growth references have since been developed for older children and adolescents, these have not been harmonized into a single internationally accepted standard [46]. This lack of a unified reference for children and adolescents older than five years, the very age group shown in this review to carry a disproportionate wasting burden, remains an unresolved barrier to consistent case detection across the continent. The failure to adopt MUAC-based screening in African pediatric oncology units likely results in the systematic under-identification of malnourished children, depriving them of timely nutritional intervention and exposing them to heightened risk of chemotherapy toxicity and mortality [18,23].

The substantial heterogeneity observed in pooled prevalence estimates (I² = 95.3%) reflects not only methodological variation but also genuine differences in the populations served by different oncology centers. Prevalence estimates ranged from 11.7% in a Kenyan cohort [35] to 67.6% in a Ethiopian cohort [39]. Some of this variation is geographic, reflecting differential burdens of baseline food insecurity across regions. South Africa, Malawi, and Ethiopia reported some of the highest wasting prevalence rates, consistent with known high burdens of child undernutrition in these settings [21,39,40,47,48]. It is also important to note that the evidence base underlying this synthesis, while pan-African in framing, is drawn from only eight of Africa’s 54 countries, with South Africa alone contributing five of the sixteen included cohorts. The findings presented here should therefore be interpreted as the best currently available evidence on paediatric oncology wasting in Africa rather than as fully representative of the continent’s diverse healthcare systems, nutritional contexts, and cancer care pathways. However, patient-level factors also contribute to heterogeneity. Older children and adolescents exhibited higher wasting prevalence than younger children in multiple cohorts [24,32], likely reflecting both the exclusion of this age group from national malnutrition screening programs and the increased metabolic demands of adolescence in the context of active malignancy. Cancer type also influences wasting risk. Gastrointestinal tumors, hematological malignancies, and advanced-stage disease were consistently associated with higher wasting prevalence, reflecting tumor-specific metabolic effects and disease severity [24,26].

Our findings regarding clinical outcomes corroborate and extend prior evidence linking malnutrition to adverse events in pediatric cancer. Across two included cohorts, wasting at diagnosis was independently associated with higher rates of chemotherapy-induced neutropenia, febrile neutropenia, sepsis, and reduced overall survival [18,23]. Poverty and household food insecurity, assessed using validated screening tools, were strong independent predictors of wasting in the South African cohort [32], reinforcing that malnutrition in the pediatric cancer population is not only a medical issue but a socioeconomic determinant of nutritional status. The same cohort additionally reported that household hunger was associated with elevated odds of treatment abandonment and mortality, suggesting that food insecurity may influence cancer outcomes through pathways beyond wasting alone. Interventions that address food insecurity at the household level, including cash transfers, food vouchers, or provision of ready-to-use therapeutic foods, may be as essential to improving survival as chemotherapy dose optimization [9,26,49,50].

The observed associations between wasting and chemotherapy toxicity merit specific attention. The Ghanaian study reported that wasted children, defined by MUAC criteria, were significantly more likely to develop severe mucositis and anemia requiring transfusion during the first 12 weeks of chemotherapy [18]. Paradoxically, the same study found that wasted children had lower rates of prolonged neutropenia than well-nourished children, possibly reflecting dose reductions or treatment delays implemented by clinicians in visibly malnourished patients. This finding shows a critical tension in resource-limited setting: the absence of standardized nutritional intervention pathways forces clinicians to make ad hoc decisions about dose modification, potentially compromising both treatment intensity and cure rates. Evidence-based guidelines for nutritional support, integrated into chemotherapy protocols, are urgently needed [6,25].

The recently published consensus recommendations for nutritional management of children with cancer in limited-resource settings, developed by the International Initiative for Pediatrics and Nutrition, provide a practical framework for implementation [9]. These adapted protocols emphasize serial MUAC measurement, structured nutritional risk stratification, and algorithm-guided interventions including oral nutritional supplements and ready-to-use therapeutic foods. Field testing in hospitals across South Africa demonstrated feasibility and acceptability, with significant improvements in nutritional status over six months of implementation [51]. However, scaling these interventions across Africa will require sustained investment, training of clinical staff, and integration of nutritional services into routine oncological care pathways. The World Health Organization has not yet recognized childhood cancer as a high-risk category warranting prioritized nutritional intervention in global malnutrition guidelines [22]. Our findings provide the epidemiological foundation to advocate for this policy change.

### Strengths and limitations

This systematic review provides the first comprehensive synthesis of wasting prevalence in African children with cancer, analyzing 16 cohorts (2,419 patients) from eight countries. Strengths include rigorous methodology using PRISMA guidelines, robust statistical pooling, and GRADE assessments, offering actionable clinical insights. However, key limitations exist. The geographic distribution of included studies was uneven, with South Africa, Malawi, and Uganda contributing the majority of cohorts and several major African regions, including North and Central Africa and most of West Africa, unrepresented; the pooled estimate should accordingly be interpreted as the best available evidence rather than a figure representative of the continent as a whole. Evidence certainty is very low due to high heterogeneity, which precluded formal subgroup meta-analyses; this heterogeneity is likely driven in part by the use of non-interchangeable nutritional assessment tools (MUAC, BMI-based indices, weight-for-height, triceps skinfold thickness, and clinical judgement) across included studies, each of which captures a distinct dimension of nutritional status. The absence of a harmonized MUAC reference standard for children older than five years further limits comparability of wasting estimates in this age group across studies. Findings rely heavily on single-center, retrospective observational studies, limiting causal inferences due to potential confounding. Furthermore, inconsistent outcome reporting prevented quantitative meta-analysis of clinical impacts.

### Implications for public health, policy, and research

Routine MUAC-based nutritional screening should become standard across all African pediatric oncology units. This cost-effective measure enables crucial early intervention and national malnutrition programs should expand eligibility beyond children under five to include all pediatric cancer patients, closing a policy gap that drives preventable mortality. From a policy perspective, global health authorities like the WHO should formally designate childhood cancer as a high-risk condition, guaranteeing automatic inclusion in therapeutic feeding programs. National treatment protocols should mandate regular nutritional assessments and establish clear clinical thresholds for interventions. We urgently need prospective randomized trials to quantify the survival benefits of structured nutritional support versus standard care. Additionally, pharmacokinetic studies are required to determine if malnutrition alters chemotherapy exposure, which could guide safer dosing strategies. Future research must also employ implementation science to sustainably integrate nutritional care into local health systems.

## Conclusions

Wasting and malnutrition affects about two in five children with cancer in the African countries studied. Wasting was independently associated with higher chemotherapy toxicity, treatment-related neutropenia, sepsis, and reduced overall survival across two of the included cohorts. The diagnostic tool employed is the single most consequential determinant of detected prevalence, and weight-based metrics alone are inadequate in populations with large solid tumours. Universal adoption of MUAC-based screening, development of a standardized and harmonized MUAC reference for children and adolescents older than five years, structured algorithm-guided nutritional intervention, and integration of socioeconomic vulnerability assessment into routine oncological care are evidence-based priorities for improving treatment tolerability and survival in this population.

## Supporting information

S1 File PRISMA checklist. (DOCX)

S2 File Database search strings. Full search strategies for PubMed, Web of Science and EMBASE.
(DOCX)

## References

[1] Allemani C, Matsuda T, Di Carlo V, Harewood R, Matz M, Nikšić M, et al. Global surveillance of trends in cancer survival 2000–14 (CONCORD-3): analysis of individual records for 37 513 025 patients diagnosed with one of 18 cancers from 322 population-based registries in 71 countries. The Lancet. 2018;391(10125):1023–75. doi: 10.1016/S0140-6736(17)33326-3 29395269 PMC5879496

[2] Santero M, Ortiz Sequeira R, Cruz Paixao M, Muñoz Martinez M, Mazorra Roig P, Chantada G, et al. Childhood cancer survival in low- and middle-income countries and the Global South: emerging evidence and critical gaps from a scoping review of observational studies. EJC Paediatr Oncol. 2025;6:None. doi: 10.1016/j.ejcped.2025.100422 41368027 PMC12682699

[3] Friedrich P, Lam CG, Kaur G, Itriago E, Ribeiro RC, Arora RS. Determinants of treatment abandonment in childhood cancer: results from a global survey. PLoS One. 2016;11(10):e0163090. doi: 10.1371/journal.pone.0163090 27736871 PMC5063311

[4] Tackling childhood cancer in sub-Saharan Africa. The Lancet Child Adolescent Health. 2022;445. doi: 10.1016/S2352-4642(22)00161-4 35716678

[5] Barr RD, Stevens MCG. The influence of nutrition on clinical outcomes in children with cancer. Pediatr Blood Cancer. 2020;67 Suppl 3:e28117. doi: 10.1002/pbc.28117 32134218

[6] Joffe L, Ladas EJ. Nutrition during childhood cancer treatment: current understanding and a path for future research. Lancet Child Adolesc Health. 2020;4(6):465–75. doi: 10.1016/S2352-4642(19)30407-9 32061318

[7] 7.WHO. WHO global initiative for childhood cancer: an overview. 2020. Accessed 2025 November 22.

[8] Lovell AL, Makamo N, Veal GJ, Bernhardt MB, Barr R, Gala RM, et al. Nutritional status, body composition and chemotherapy dosing in children and young people with cancer: a systematic review by the SIOP nutrition network. Br J Cancer. 2025;133(3):275–85. doi: 10.1038/s41416-025-03023-3 40571762 PMC12322071

[9] Viani K, Alves J, Damasco-Avila E, Murra MS, Schoeman J, Walters M. Consensus recommendations for the nutritional management of children with cancer in limited resource settings: a report from the International Initiative for Pediatrics and Nutrition. Frontiers Media SA; 2025. doi: 10.3389/fnut.2025.1605632 PMC12240747 40642169

[10] 10.WHO. Child growth monitoring: a technical guide. 2024. Accessed 2026 April 11.

[11] Schoeman J, Kellerman I-M, Rogers PC, Ladas EJ, Lombard CJ, Uys R, et al. Prevalence of vitamin and iron deficiencies at cancer diagnosis at two pediatric oncology units in South Africa. Pediatr Hematol Oncol. 2023;40(8):752–65. doi: 10.1080/08880018.2023.2188920 36940097

[12] Schoeman J, Kellerman I-M, Ndlovu S, Ladas EJ, Rogers PC, Lombard CJ, et al. Prevalence of poverty and hunger at cancer diagnosis and its association with malnutrition and overall survival in South Africa. Nutr Cancer. 2023;75(7):1551–9. doi: 10.1080/01635581.2023.2214970 37227249

[13] Arends J, Bachmann P, Baracos V, Barthelemy N, Bertz H, Bozzetti F, et al. ESPEN guidelines on nutrition in cancer patients. Clin Nutr. 2017;36(1):11–48. doi: 10.1016/j.clnu.2016.07.015 27637832

[14] Barreto R, Waning DL, Gao H, Liu Y, Zimmers TA, Bonetto A. Chemotherapy-related cachexia is associated with mitochondrial depletion and the activation of ERK1/2 and p38 MAPKs. 2016;7. 10.18632/oncotarget.9779 PMC5190036 27259276

[15] Setiawan T, Sari IN, Wijaya YT, Julianto NM, Muhammad JA, Lee H, et al. Cancer cachexia: molecular mechanisms and treatment strategies. J Hematol Oncol. 2023;16(1):54. doi: 10.1186/s13045-023-01454-0 37217930 PMC10204324

[16] Ni J, Zhang L. Cancer cachexia: definition, staging, and emerging treatments. Cancer Manag Res. 2020;12:5597–605. doi: 10.2147/CMAR.S261585 32753972 PMC7358070

[17] Triarico S, Rinninella E, Cintoni M, Capozza MA, Mastrangelo S, Mele MC. Impact of malnutrition on survival and infections among pediatric patients with cancer: a retrospective study. J Pediatric Hematol Oncol. 2023. 10.26355/eurrev_201901_17009 30779086

[18] Salifu N, Segbefia CI, Tette EMA, Alhassan Y, Renner LA. Nutritional status at diagnosis of childhood cancer in Korle Bu Teaching Hospital, Accra, Ghana. Health Sci Investigat J. 2022;3(2):365–73. doi: 10.46829/hsijournal.2022.12.3.2.365-373

[19] Pedretti L, Massa S, Leardini D, Muratore E, Rahman S, Pession A, et al. Role of nutrition in pediatric patients with cancer. Nutrients. 2023;15(3):710. doi: 10.3390/nu15030710 36771416 PMC9920596

[20] Uzma S, Mansoor R, Jamal B, Khan L. Impact of malnutrition on survival and treatment-related morbidity of cancer in children. J Health Rehabili Res. 2024;4(2):696–701. doi: 10.61919/jhrr.v4i2.899

[21] Huibers MHW, Manda G, Silverstein A, Wanda W, Mtete I, Makuti S, et al. The burden of malnutrition in childhood cancer in Malawi – risk regardless of age. Nutr Cancer. 2022;74(9):3322–8. doi: 10.1080/01635581.2022.2076888 35608604

[22] Makamo N, Schoon S, Ozuah N, Kaspers G, Ladas EJ, Huibers M. Prevalence of undernutrition in children with cancer in low-income and middle-income countries: a systematic review. BMJ Glob Health. 2025;10(6):e019345. doi: 10.1136/bmjgh-2025-019345 40541281 PMC12182048

[23] Nyeko R, van Heerden J, Kambugu JB, Geriga F, Angom R, de Rojas T, et al. Wasting and short-term outcomes among children with cancer in resource-limited settings: a prospective study in Uganda. PLoS One. 2025;20(8):e0330107. doi: 10.1371/journal.pone.0330107 40773483 PMC12331082

[24] Wannyana D, Bagonza A, Mwima SJ, Nalwadda C, Ndejjo R. Factors associated with wasting among pediatric cancer patients aged 2-17 years at Uganda cancer institute: a cross-sectional study. PLoS One. 2025;20(9):e0333076. doi: 10.1371/journal.pone.0333076 41004519 PMC12469180

[25] Viani K, Trehan A, Manzoli B, Schoeman J. Assessment of nutritional status in children with cancer: a narrative review. Pediatr Blood Cancer. 2020;67 Suppl 3:e28211. doi: 10.1002/pbc.28211 32096326

[26] Katabalo DM, Raveendran Y, Liwa AC, Kidenya BR, Schroeder K. Nutritional status and barriers to optimal nutrition among pediatric patients with cancer in Tanzania: a quantitative analysis. J Health Popul Nutr. 2025;44(1). doi: 10.1186/s41043-025-00931-1 40474244 PMC12139128

[27] Geddara N, Mubaiwa L, Thejpal R, Hendricks C. Prevalence of malnutrition and its impact on outcomes in children with cancer in a South African setting. S Afr J Child Health. 2023;:181–5. doi: 10.7196/sajch.2023.v17i4.1986

[28] Moher D, Shamseer L, Clarke M, Ghersi D, Liberati A, Petticrew M, et al. Preferred reporting items for systematic review and meta-analysis protocols (PRISMA-P) 2015 statement. Syst Rev. 2015;4(1):1. doi: 10.1186/2046-4053-4-1 25554246 PMC4320440

[29] Pitua I, Wannyana D, Abila DB, Bongomin F. Prevalence, diagnostic methods, and clinical outcomes of wasting/cachexia among pediatric cancer patients in Africa: a protocol for a systematic review and meta-analysis. PLoS One. 2026;21(5):e0349113. doi: 10.1371/journal.pone.0349113 42102058 PMC13155595

[30] Institute JB. Checklist for randomized controlled trials. 2017. http://joannabriggs.org/research/critical-appraisal-tools.html

[31] Guyatt GH, Oxman AD, Vist GE, Kunz R, Falck-Ytter Y, Alonso-Coello P, et al. GRADE: an emerging consensus on rating quality of evidence and strength of recommendations. BMJ. 2008;336(7650):924–6. doi: 10.1136/bmj.39489.470347.AD 18436948 PMC2335261

[32] Schoeman J, Kellerman I, Ndlovu S, Ladas EJ, Rogers PC, Naidu G, et al. Prevalence of chronic and acute malnutrition and association with overall three‐year survival in newly diagnosed children with cancer in South Africa. J Human Nutrition Diet. 2025;38(4). doi: 10.1111/jhn.70082 40613404

[33] Israëls T, Chirambo C, Caron HN, Molyneux EM. Nutritional status at admission of children with cancer in Malawi. Pediatr Blood Cancer. 2008;51(5):626–8. doi: 10.1002/pbc.21697 18668514

[34] Wilde JCH, Lameris W, van Hasselt EH, Molyneux EM, Heij HA, Borgstein EG. Challenges and outcome of Wilms’ tumour management in a resource-constrained setting. Afr J Paediatr Surg. 2010;7(3):159–62. doi: 10.4103/0189-6725.70416 20859020

[35] Shikuri R, Waudo J, Kuria E. Factors influencing nutritional status and food consumption patterns of children with cancer: a case of Kenyatta National Hospital, Kenya. 2017.

[36] Geel JA, Eyal KC, Hendricks MG, Myezo KH, Stones DK, Omar F, et al. Prognostic factors affecting survival in children and adolescents with HIV and Hodgkin lymphoma in South Africa. Leuk Lymphoma. 2021;62(12):2854–63. doi: 10.1080/10428194.2020.1852472 33284043

[37] Draper KS, Hadley G, Pillay K, Wiles NL. Relationship between nutritional status and treatment-related neutropenia in children with nephroblastoma. South African J Clinical Nutrition. 2017;31(4):74–7. doi: 10.1080/16070658.2017.1401289

[38] Tola WO, Melaku T, Fufa D, Sheleme T. Adverse drug events and contributing factors among pediatric cancer patients at Jimma University medical center, Southwest Ethiopia. BMC Pediatrics. 2023;23(1):77. doi: 10.1186/s12887-023-03891-9 36782170 PMC9923905

[39] Yifru S, Muluye D. Childhood cancer in Gondar University Hospital, Northwest Ethiopia. BMC Res Notes. 2015;8(1). doi: 10.1186/s13104-015-1440-1 26404043 PMC4582631

[40] Lifson LF, Hadley GP, Wiles NL, Pillay K. Nutritional status of children with Wilms’ tumour on admission to a South African hospital and its influence on outcome. Pediatr Blood Cancer. 2017;64(7):10.1002/pbc.26382. doi: 10.1002/pbc.26382 28027433

[41] Tazi I, Hidane Z, Zafad S, Harif M, Benchekroun S, Ribeiro R. Nutritional status at diagnosis of children with malignancies in Casablanca. Pediatr Blood Cancer. 2008;51(4):495–8. doi: 10.1002/pbc.21689 18636463 PMC4684256

[42] Zimmermann K, Ammann RA, Kuehni CE, De Geest S, Cignacco E. Malnutrition in pediatric patients with cancer at diagnosis and throughout therapy: a multicenter cohort study. Pediatr Blood Cancer. 2013;60(4):642–9. doi: 10.1002/pbc.24409 23281136

[43] Revuelta Iniesta R, Paciarotti I, Davidson I, McKenzie JM, Brougham MFH, Wilson DC. Nutritional status of children and adolescents with cancer in Scotland: a prospective cohort study. Clin Nutr ESPEN. 2019;32:96–106. doi: 10.1016/j.clnesp.2019.04.006 31221298

[44] Iniesta RR, Paciarotti I, Brougham MFH, McKenzie JM, Wilson DC. Effects of pediatric cancer and its treatment on nutritional status: a systematic review. Nutr Rev. 2015;73(5):276–95. doi: 10.1093/nutrit/nuu062 26011902

[45] Israëls T, Borgstein E, Jamali M, de Kraker J, Caron HN, Molyneux EM. Acute malnutrition is common in Malawian patients with a Wilms tumour: a role for peanut butter. Pediatr Blood Cancer. 2009;53(7):1221–6. doi: 10.1002/pbc.22158 19821536

[46] Mramba L, Ngari M, Mwangome M, Muchai L, Bauni E, Walker AS, et al. A growth reference for mid upper arm circumference for age among school age children and adolescents, and validation for mortality: growth curve construction and longitudinal cohort study. BMJ. 2017;358:j3423. doi: 10.1136/bmj.j3423 PMC5541507 28774873

[47] Riwa FP, Odgers-Jewell K, Jones MA, Mushi AA. The prevalence and determinants of undernutrition among infants and children aged 6 months to 5 years in sub-Saharan African countries: a systematic scoping review. Nutr Rev. 2025;83(7):e1896-916. doi: 10.1093/nutrit/nuae189 PMC12166183 39760760

[48] Marume A, Kasanzu S, Chirenda J. Socio-economic and rural-urban disparities in the double burden of childhood malnutrition in sub-Saharan Africa. J Health Popul Nutr. 2025;44(1):335. doi: 10.1186/s41043-025-01075-y 41029882 PMC12487389

[49] Pedretti L, Massa S, Leardini D, Muratore E, Rahman S, Pession A, et al. Role of nutrition in pediatric patients with cancer. Nutrients. 2023;15(3):710. doi: 10.3390/nu15030710 36771416 PMC9920596

[50] Durao S, Visser ME, Ramokolo V, Oliveira JM, Schmidt B-M, Balakrishna Y, et al. Community-level interventions for improving access to food in low- and middle-income countries. Cochrane Database Syst Rev. 2020;8(8):CD011504. doi: 10.1002/14651858.CD011504.pub3 32761615 PMC8890130

[51] Schoeman J, Kellerman I-M, Ladas EJ, Ndlovu S, Rogers PC, du Plessis J, et al. Implemented nutritional intervention algorithm in pediatric oncology compared to standard nutritional supportive care outcomes. Clin Nutr ESPEN. 2024;63:870–7. doi: 10.1016/j.clnesp.2024.08.019 39197726

